# Giant intrapulmonary solitary fibrous tumor

**DOI:** 10.4322/acr.2024.494

**Published:** 2024-06-21

**Authors:** Sheela Devi Chandakavadi Shivalingaiah, Deepika Gurumurthy, Gauri Dadich

**Affiliations:** 1 Jagadguru Sri Shivarathreeshwara Academy of Higher Education & Research (JSSAHER), Department of Pathology, Mysore, Karnataka, India.

**Keywords:** Solitary Fibrous Tumors, Lung, Immunohistochemistry

## Abstract

Solitary fibrous tumor (SFT) is a soft tissue tumor of mesenchymal origin involving, most commonly, the pleura. Intrapulmonary SFT is a slow-growing tumor that rarely reaches giant forms. SFTs are asymptomatic and often randomly discovered by routine chest X-rays. The diagnosis requires histopathological and immunohistochemical (IHC) examinations. Most of the SFTs are benign and present an indolent course. Larger tumors are more likely to be malignant and consequently associated with a worse prognosis. Despite having histopathological criteria for malignancy, the behavior of SFTs is challenging to predict. We report a case of giant intrapulmonary SFT of intermediate risk.

## INTRODUCTION

Solitary fibrous tumor (SFT) is a soft tissue tumor of mesenchymal origin that most commonly involves the pleura.^1,2^ SFT also occurs in other sites such as the lung parenchyma, pericardium, pelvis, abdomen, retroperitoneum, buccal space, maxillary sinus, liver, pancreas, suprarenal region, and kidneys.^3^ It is a slow-growing neoplasm, and giant forms are rarely encountered.^4^ SFT is considered “giant” when the diameter is greater than 15 cm or when tumors occupy more than 40% of the hemithorax.^5^ The estimated SFT incidence is less than 2% of all soft tissue tumors, approximately 0.2 per 100,000 people annually.^2^ The peak age of incidence is between the 5^th^ and 7^th^ decade of life, although it is seen in all ages with no gender predisposition.^6,7^ In their early stage, SFTs are asymptomatic and are often randomly discovered by routine chest X-rays.^4^ These tumors tend to grow into massive lesions before any evidence of local compression symptoms like cough, dyspnea, and chest pain.^4,6^Rarely, hypertrophic pulmonary osteoarthropathy and hypoglycemia may accompany the clinical presentation due to the abnormal production of hyaluronic acid and insulin-like growth factor-2 by the tumor. The presence of pleural effusion and compressive symptoms are more likely in patients with malignancy and large tumors.^8^ Compared to mesothelioma, there is no association with exposure to tobacco, asbestos, or other environmental agents.^6,7^ The cell of origin of SFTs is believed to be the subpleural mesenchymal cell. These CD34-positive tumor cells are now considered to be ‘‘dendritic interstitial cells’’ with antigen-presenting ability.^9^ Diagnosis of extra pleural SFT is challenging and requires clinical, histological, immunohistochemical, and even molecular findings.^10^ We report a case of giant intrapulmonary SFT.

## CASE REPORT

A 60-year-old man, a railway employee, presented with complaints of loss of appetite and loss of weight over the last year. He started presenting fever and chills over the previous week, followed by shortness of breath. He is a chronic alcoholic and smoker with Type-2 diabetes mellitus.

The thoracic ultrasonography showed left pleural effusion, underlying lung consolidation/collapse, and a heteroechoic mass lesion within the left lung. His thoracic contrasted enhanced computed tomography showed a well-defined heterogeneously enhancing soft tissue density mass with a large central hypodense area in the left hemithorax, with collapse of the left lung segments. Wide local excision with left lower anterior segmentectomy was performed.

**Figure 1:**
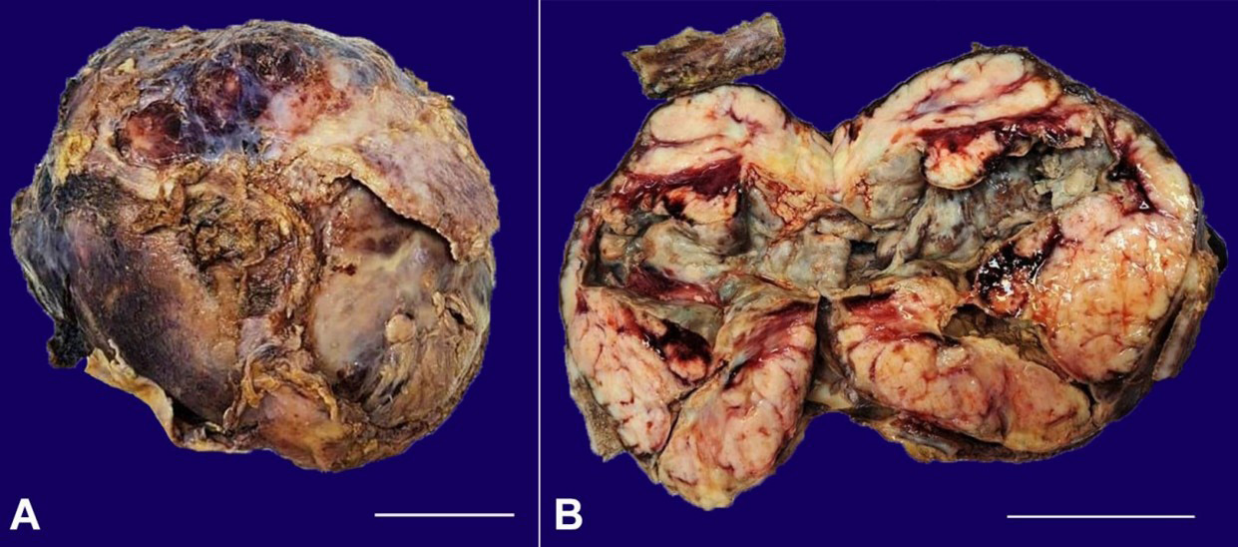
Gross view of the surgical specimen. **A –** Large grey-white mass measuring 17.5 ×15 ×9 cm (scale bar= 5 cm); **B –** Large grey-white mass with grey -yellow areas and hemorrhage in the left lung, measuring 17.5 ×15 ×9 cm with a segment of rib measuring 4x2x1cm (scale bar= 5 cm).

We received a specimen of left lung lower anterior segmentectomy measuring 18×15×9.6 cm and weighing 1.5 kg. On the cut section, a large grey-white tumor was seen occupying the whole segment, measuring 17.5 ×15 ×9 cm with grey-yellow areas and hemorrhage (Figure 1A, 1B). We also received a segment of rib measuring 4x2x1cm.

**Figure 2:**
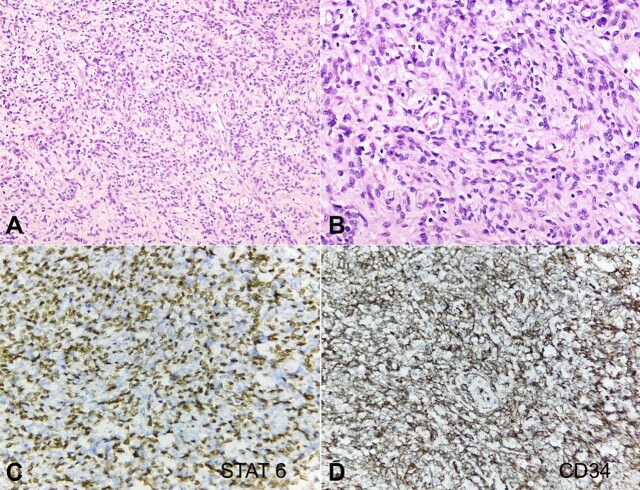
Photomicrographs of the tumor. **A** and **B –** Spindle cells with fusiform nucleus arranged in fascicles, sheets, hemangiopericytomatous and pattern-less patterns with collagenous stroma. (H&E, x200, x400 respectively); **C –** STAT6 strongly positive reaction (x400); **D –** CD34 positive reaction (x400).

Microscopically, the tumor was comprised of spindle cells with a fusiform nucleus arranged in bundles, sheets, hemangiopericytomatous and pattern-less patterns with collagenous stroma (Figures 2A and 2B). Mitotic figures were 1-2 per 10 high-power fields. Focal areas of necrosis and hemorrhage were present.

On immunohistochemistry, the tumor cells were diffusely and strongly positive for STAT6, BCL2, and CD34 (Figure 2C and 2D). MiB1/Ki67 was 10-12%. The final diagnosis of a solitary fibrous tumor of intermediate risk was made.

The immediate postoperative course was uneventful. The patient has yet to turn up for the follow-up visit. According to the care-takers, the patient is clinically doing well at the end of 1-year post-surgery follow-up.

## DISCUSSION

SFT arises most commonly from the pleura. But SFT occurring within the lung parenchyma i.e., intrapulmonary SFT, is a poorly recognized entity since it has rarely been reported.^11,12^ According to a study by Lin et al.,^12^ around 45 patients with intrapulmonary SFTs were retrieved by searching databases, and 4 cases out of them were giant intrapulmonary SFTs, the largest of 22 cm in its longest axis. However, they excluded some studies due to the lack of information. The preoperative diagnosis is difficult because of non-specific clinical and imaging findings. Hence, the correct diagnosis requires pathological and immunohistochemical examination of the resected surgical specimens.^1^

The results of the studies are contradictory concerning the association between tumor size and the risk of recurrence. While some large tumors are more likely to be malignant and are associated with a worse prognosis,^13^ others suggest that size is not a risk factor for recurrence and long-term survival is excellent.^14^

It is difficult to predict the biological behavior of SFTs. Histologically benign tumors may still recur.^2^ The malignant behavior of SFT may be suspected if the following features are seen: hypercellularity, pleomorphism, tumor necrosis and hemorrhage, > 4 mitoses/10 high-power fields, and tumor size of 10cm or larger.^13^ Diebold et al.,^15^ have noted that a high proliferation index MIB-1 of more than 10% was independently associated with adverse outcomes. In our case, the tumor was 17.5cm with 1-2 mitotic figures/10 hpf, focal areas of necrosis, hemorrhage, and MiB1/Ki67 was 10-12%. Hence, the diagnosis of a solitary fibrous tumor of intermediate risk was made.

The differential diagnosis includes pulmonary adenofibroma, synovial sarcoma, benign neural neoplasms, leiomyoma, leiomyosarcoma, spindle cell carcinoid tumor, nerve sheath tumor, spindle cell thymoma, fibrosarcoma, sarcomatoid carcinoma, and sarcomatoid mesothelioma. IHC plays a significant role in making a definitive diagnosis.^12^ IHC findings in intrapulmonary SFT are similar to those in pleural SFT.^16,17^ The most important positive markers in SFT are CD34, Bcl-2, STAT-6 which usually show diffuse and strong expression.^16,18^ Expression of CD34 has been observed in 81–95% of SFTs but is lost in malignant tumors. BCL-2 is a sensitive marker (> 90% sensitivity) with low specificity. Although a distinct IHC profile is expressed in SFT, the classic histological and IHC profile is not seen in all cases and diagnosis can be challenging.^18^ NAB-STAT6 gene fusion has been shown to be present in the majority of SFT. STAT6 IHC stain has emerged as a useful surrogate marker of NAB2-STAT6 gene fusion with a sensitivity and specificity of 98% and 85%, respectively. It is also expressed in malignant cases.^18^ It helps to diagnose rare neoplasms and to distinguish them from histologic mimics.^15^ In our case, tumor cells were diffusely and strongly positive for STAT6, BCL2, and CD34.

The mainstay of treatment for all benign and malignant SFT is complete tumor resection with a recommendation of a 1 to 2-cm margin of healthy tissue.^17^ Complete excision of giant SFT may be a challenging task.^4^ Intraoperative bleeding is a major complication, especially with giant tumors, and preoperative embolization may be helpful.^5^ The tumor’s behavior does not always correlate with histologic findings. Rao et al.^11^ have reported recurrence in 20% of benign cases of intrapulmonary SFT. Also, late recurrence after more than 20 years has been reported after the initial resection.^17^ Hence, it is crucial to have a long-term follow-up of all the patients.

Given the rare occurrence of intrapulmonary localized SFT of the lung, more studies are needed to clarify their clinicopathologic behavior.

## CONCLUSION

Intrapulmonary SFT is a relatively uncommon tumor, and accurate diagnosis is essential for appropriate management. Giant SFTs deserve special consideration since they may be associated with various clinical symptoms, technically challenging surgery, and unpredictable clinical outcomes. STAT6 IHC stain has emerged as a useful surrogate marker of NAB2-STAT6 gene fusion with excellent sensitivity and specificity, which is practical and economically feasible. Extensive sampling is required to exclude malignancy. It is crucial to have long-term patients’ follow-ups because of the difficulty in predicting the future biological behavior of the tumors.
